# Opportunities and Challenges of Soy Proteins with Different Processing Applications

**DOI:** 10.3390/antiox13050569

**Published:** 2024-05-05

**Authors:** Zixiao Deng, Sung Woo Kim

**Affiliations:** Department of Animal Science, North Carolina State University, Raleigh, NC 27695, USA; zdeng7@ncsu.edu

**Keywords:** antinutritional compounds, bioactive compounds, processed soy product, soybean meal

## Abstract

Soybean meal (SBM) is a prevailing plant protein supplement in animal diets because of its nutritional value and availability. This review paper explores the significance of SBM and processed soy products, emphasizing their nutritional and bioactive components, such as isoflavones and soyasaponins. These compounds are known for their antioxidant and anti-inflammatory properties and are associated with a reduced prevalence of chronic diseases. However, the presence of antinutritional compounds in SBM presents a significant challenge. The paper evaluates various processing methods, including ethanol/acid wash, enzyme treatment, and fermentation, which are aimed at enhancing the nutritional value of soy products. It highlights the significance to maintain a balance between nutritional enhancement and the preservation of beneficial bioactive compounds, emphasizing the importance of different processing techniques to fully exploit the health benefits of soy-based products. Therefore, this review illuminates the complex balance between nutritional improvement, bioactive compound preservation, and the overall health implications of soy products.

## 1. Introduction

Soybean (*Glycine max* [L.] Merrill) stands as a pivotal and indispensable crop on the global agricultural stage. In 2021, the global soybean production was 371.7 million tons and Brazil (134.9 million tons), the United States of America (120.7 million tons), and Argentina (46.2 tons) contributed around 82% of the total soybean production [1,2]. Due to the rapid increase in population in developed countries, animal protein consumption keeps increasing [3,4]. In 2012, 155 million tons of feed protein were consumed by monogastric animals, and the consumption was estimated to be 207 million tons in 2030 [5]. Soybean meal is a major co-product of the extraction of oil for soybean, and the residue after extraction is rich in protein. Since the early 20th century, the production of SBM has experienced consistent growth over time. Globally, SBM production surged from 216 million tons in 2015 to 240 million tons in 2019 [6]. In 2023, the United States exported 14 million tons of SBM, valued at 7.38 billion dollars, to other countries. This trend had been consistently increasing by 3% annually since 2014 [7].

Roughly 97% of the total soybean meal production serves as a vital ingredient in the animal feed industry, while the remaining 3% is dedicated to human consumption in various forms such as protein alternatives, soymilk, and meat analogs, among others [8]. Soybean meal is the major source of amino acids in poultry and pig production globally [2]. Soybean meal also serves as a significant source of metabolizable and net energy [6]. There is an increasing use of soybean meal in companion animal food sectors and rather recently, soybean meal became a significant protein source in aquaculture feeds [9]. Soy protein products for human consumption are primarily found in the form of isolated soy protein (ISP). The ISP is the most concentrated form of commercially available soybean protein and contains a minimum protein content of 90% on a dry matter basis [10]. Although manufacturing process of ISP has slight variations according to the manufacturer, the overall objective remains consistent: to separate the fiber (pectin, cellulose, and hemicellulose) and carbohydrate (sucrose and various oligosaccharides) from the protein. A common method involves the extraction of white flakes using alkaline water (pH 8 to 9) to effectively segregate the protein and soluble carbohydrates from fibrous materials [11].

Soybean meal is considered the most common plant-based protein supplement for animal diets due to its high-quality nutrients [12]. Numerous factors, including soybean genotype, geographical region, soil composition, agricultural techniques, climate, and processing conditions, collectively influence the chemical compositions and, consequently, the nutritional profiles of soybean meal [13]. The major storage protein in soybeans is globulins, which makes up approximately 90% of this category. The remaining portion consists of albumins. In the category of globulins, there is 36 to 53% glycinin (11S), 30 to 46% β-conglycinin (7S), 13 to 18% 2S, and 0 to 4% 15S [14]. Beta-conglycinin comprises three subunits including α (67 kDa), α′ (71 kDa), and β (50 kDa); glycinin is a hexamer and comprises acidic subunit A (35 kDa) and basic subunit B (20 kDa) that were linked by a disulfide bond [15,16,17]. Fraction 2S consists of Bowman–Birk- and Kunitz-type trypsin inhibitors, cytochrome c and α-conglycinin [14]. After oil extraction, the crude protein content of SBM varies between 41.0 to 50.0% on a dry basis, with fluctuations attributed to the quantity of hulls present and the specific processing method employed [18]. Carbohydrates constitute the second most prominent group in SBM ranging from 35% to 40% [19]. It is primarily composed of non-starch polysaccharides (NSP, 17% pectins, 8% cellulose), free sugars (5% sucrose, 4% stachyose, 1% raffinose), and few starch (<1%) [20].

Soybean meal contains high contents of bioactive compounds, mainly isoflavones and soyasaponins, which play important roles in human and animal health [21,22]. These bioactive compounds have been demonstrated to exhibit antioxidant, anti-inflammatory, and antiviral properties across a diverse range of target cell populations [23]. Furthermore, they are linked to a reduced incidence of several chronic conditions, notably cardiovascular diseases and specific forms of cancer [24]. Despite the high nutritional values and bioactive compounds, the antinutritional compounds in SBM limit the application in animal feed, especially for young animals [25,26]. To reduce the antinutritional compounds in soybean meal or soy flakes and improve the nutritional value, several methods were established including ethanol/acid wash, enzyme treatment, and fermentation, which ended up with soy protein concentrate (SPC) [27,28], enzyme-treated soybean meal (ESBM) [29,30], and fermented soybean meal (FSBM) [17,31]. The elimination of oligosaccharides, as well as glycinin and β-conglycinin from SBM/soybean flakes, significantly enhances their nutritional value [32,33]. However, there are some potential risks to reduce bioactive compounds as those methods are implemented. Therefore, this review paper focuses on an analysis of the advantages and disadvantages associated with SBM and various processed soy products.

## 2. Bioactive Compounds in Soybean Meal

Isoflavones, predominantly located in soybeans, soy foods, and legumes [34,35], exhibit phytoestrogenic effects by mimicking hormonal activity through their attachment to estrogen receptors (ER) in mammals [36]. Due to their structural similarities to 17β-estradiol, it allows isoflavones to function as either weak agonists or antagonists of natural estrogen, contingent on the specific cellular concentration [37]. Therefore, the modulation of steroid receptors by isoflavones is dependent upon both dosage and duration of action, which can manifest as either transient and remarkably swift or sustained effects [38]. Studies have shown that isoflavones have the capability to down-regulate mRNA for aromatase in human granulosa-luteal cells, potentially influencing steroidogenesis [39]. Estrogens can show the genomic effects mainly through two nuclear receptors, estrogen ERα and ERβ [40]. Analysis of relative molar binding affinities of various estrogenic compounds suggests that phytoestrogens exhibit higher affinities for ERβ, suggesting that this receptor subtype may play a more important role in the actions of non-steroidal estrogens [41]. Among these isoflavones in soybeans, genistein and daidzein are the most prevalent isoflavones in soybeans. Initially present in glycoside forms which are not bioavailable, these isoflavones require transformation within the digestive tract into their aglycone forms, a process facilitated by β-glycosidases [42]. Beta-glycosidases, functioning as brush border enzymes in the small intestine, are also significantly present as microbial enzymes within the hindgut of the gastrointestinal tract in monogastric species [43]. Following their hydrolysis, genistein and daidzein are either directly assimilated in the intestine or further metabolized through hydrogenation into a variety of other bioactive compounds, including equol, 5,7,4′-rihydroxyisoflavan, 4,7,4′-trihydroxyflavan, dihydrodiadzein (DHD), dihydrogenistein (DHG), and others [44]. Isoflavone effects on pregnant animals exhibit variability. Research in rodent models indicates that administering genistein, bisphenol, and other environmental estrogenic compounds in females disrupted embryo implantation in adult female offspring, potentially due to oviduct–uterine abnormalities [45,46]. Regarding the impact of soy isoflavones on the blastocyst, isoflavone-induced inhibition of glucose uptake could inhibit early embryonic development, as glucose serves as the primary source of exogenous energy during the initial stages of pre-implantation embryo development [47]. Furthermore, high concentrations of genistein have been shown to inhibit phosphorylation of tyrosine in cadherin–catenin complexes, which play crucial roles in compaction, adhesive functions, and embryonic cleavage in mouse embryos [48]. Moreover, genistein injection during late gestation has demonstrated positive effects on insulin-like growth factor 1 (IGF1) levels in pigs, whilst not affecting fetal growth and development [49].

Soyasaponins, also present in soybeans and legumes like lentils and green peas, are characterized as amphiphilic oleanane triterpenoid glycosides with polar sugar chains conjugated to a nonpolar pentacyclic ring and are classified into four major groups based on their aglycones: groups A, B, E, and DDMP (2,3-dihydro-2,5-dihydroxy-6-methyl-4H-pyran-4-one) [50]. The processed forms of soybeans often contain soyasapogenols A, B, and E, which are derived from the acid or alkaline hydrolysis of soyasaponins and are not naturally occurring in raw soybeans. Other derivatives, soyasapogenols C and D, can emerge from the acid hydrolysis of soyasapogenol B, representing non-native aglycones of soyasaponins [51,52]. However, the bioavailability of soyasaponins is still not clear and requires further investigation. Direct absorption of soyasaponins in animals is very limited due to the chemical structure and properties (large molecular size and amphiphilic compounds) [53]. Most soyasaponins would be degraded by intestinal microbiota and release sugars and aglycones [54].

Free radicals and reactive oxygen species (ROS) are byproducts of normal oxygen metabolism and can also be stimulated by external factors, such as phagocytosis [55]. When produced in excess, these reactive molecules can lead to harmful reactions including the peroxidation of membrane lipids, oxidative damage to nucleic acids and carbohydrates, and the oxidation of vulnerable protein groups [56,57]. Typically, ROS triggers the release of various inflammatory mediators, attracting neutrophils and other inflammatory cells, thereby promoting inflammation and tissue damage [58]. Isoflavones have been demonstrated to enhance the expression of antioxidant genes, such as the cystine/glutamate anionic amino acid transporter (xCT), glutamate cystine ligase (GCL), glutathione reductase (GR), heme oxygenase-1 (HO-1), NAD(P)H-quinone oxidoreductase-1 (NQO1) and sequestosome-1 (SQSTM1) [59,60]. These compounds also activate kinases, such as JNK, p38, PI3K and Akt which may lead to the phosphorylation of Nrf2, enhancing its accumulation in the nucleus. Concurrently, the phosphorylation of Bach1 facilitates its removal from the nucleus, further promoting Nrf2 binding to antioxidant response elements (ARE) [61]. This upregulation of antioxidant gene expression plays a pivotal role in restoring the redox balance and mitigating oxidative damage in the host.

Isoflavones exert their anti-inflammatory effects primarily by inhibiting the production of pro-inflammatory cytokines and chemokines, such as IL-1β, IL-6, IL-12, and tumor necrosis factor-α (TNF-α) [62]. During an inflammatory response, nuclear factor kappa-light-chain-enhancer B cells (NF-κB), located in the cytoplasm, are activated by IκB kinase (IKK). This activation leads to the translocation of NF-κB into the nucleus, where it stimulates the transcription of genes responsible for the production of pro-inflammatory agents, including cytokines, chemokines, inducible nitric oxide synthases (iNOS), and cyclooxygenase 2 (COX-2) [63]. Isoflavones can suppress the production of these inflammatory molecules by inhibiting the NF-κB transcriptional pathway [62]. Additionally, they modulate the metabolism of arachidonic acid (AA) and the production of nitric oxide (NO) by reducing the levels and activities of various pro-inflammatory enzymes, such as phospholipase A2 (PLA2), lipoxygenase (LOX), COX-2, and inducible nitric oxide synthases (iNOS) [64,65,66,67].

Soyasaponins demonstrate a significant ability to suppress the production of pro-inflammatory cytokine TNF-α and chemokine monocyte chemoattractant protein-1 (MCP-1), as well as key inflammatory mediators such as prostaglandin E2 (PGE2) and NO. They also inhibit inflammatory enzymes including cyclooxygenase-2 (COX-2) and inducible nitric oxide synthase (iNOS), and prevent the degradation of IκB-α, an inhibitor of the nuclear transcription factor kappa B (NF-κB), in lipopolysaccharide (LPS)-stimulated macrophages [68]. Additionally, soyasaponin Ab obstructs the attachment of LPS and Alexa-Fluor-594-conjugated LPS to Toll-like receptor 4 (TLR4) on macrophages, a key receptor associated with the NF-κB pathway through IL-1 receptor-associated kinases (IRAKs) and it is responsible for recognizing pathogen-associated molecular patterns like LPS [69].

## 3. Antinutritional Compounds in Soybean Meal

**Figure 1:**
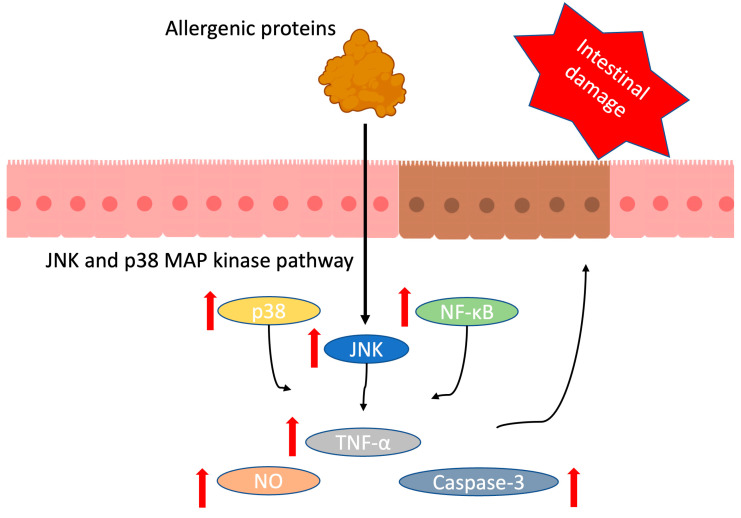
Pathway of soybean glycinin- and β-conglycinin-induced intestinal damage in animals. Glycinin (11S) and β-conglycinin (7S) increased mRNA and protein expression of jun N-terminal kinase (JNK), p38, and nuclear factor-kappa B (NF- κB). Soy antigens enhanced the level of nitric oxide (NO), tumor necrosis factor-α (TNF- α), and caspase-3, resulting in intestinal damage [77]. The red upward arrow represents the increase of the corresponding indicator.

The main antinutritional compounds in SBM include allergenic proteins, trypsin inhibitors (TI), goitrogens, lectins, mineral binding compounds, and several other detrimental factors [70]. Due to the transient hypersensitivity caused by soy antigens, young animals may have damaged intestinal morphology, increased immune response, and impaired growth performance [26]. Glycinin and β-conglycinin, two major storage proteins in soybean, have been identified as important allergenic sources [71,72]. The allergic reaction may be different according to species, age, and breed, but generally it appears in young animals [73,74]. Glycinin could stimulate local and systemic immune responses of young animals by increasing lymphocyte proliferation, CD4+/CD8+ ratio, intestinal immunoglobulin A (IgA), interleukin-4 (IL-4) and IL-6, which has negative effects on growth performance [75]. Beta-conglycinin could stimulate an intrinsic immune response and induce an allergic reaction in animals, which was mediated by IgE. And it causes intestinal damage, higher level of IL-4, interferon-γ (INF-γ), and histamine, which may be the result of T helper1 and T helper2 responses [76]. The mechanism of soybean glycinin- and β-conglycinin- induced intestinal damage was investigated (Figure 1) [77]. Mitogen-activated protein kinases (MAPKs) control a wide range of cellular functions, including development, metabolism, apoptosis, and innate immune responses [78,79,80]. Jun N-terminal kinase (JNK) and p38, both belonging to the MAPK family, are recognized subgroups known for their significant involvement in regulating cell apoptosis and proliferation [81]. The transcription factor, nuclear factor-kappa B (NF-κB), is an important component of the immune system that controls the expression of cytokines, growth factors, and effector enzymes in response to the ligation of several immune receptors [82]. Glycinin and β-conglycinin could increase JNK, p38, and NF-κB mRNA and protein expression, and their phosphorylation levels. In addition, soy antigens could enhance the level of nitric oxide (NO), tumor necrosis factor-α, and caspase-3, resulting in intestinal damage in animals [77].

Soybean meals are rich in carbohydrates, primarily composed of non-starch polysaccharides (NSP) and oligosaccharides. The NSP digestion primarily involves acidic breakdown in the stomach and subsequent microbial fermentation that occurs predominantly in the distal part of the small intestine and throughout the entire large intestine [20]. However, the soluble fraction of NSP could lead to decreased nutrient digestion and absorption in monogastric animals [83,84], which resulted from increased digesta viscosity, including the changes in intestinal physiology and the overall intestinal ecosystem [85]. In addition, the soluble NSP was positively correlated with pathogenesis of dysentery [86]. Stachyose and raffinose are considered two main oligosaccharides in the soybean meal that result in intestinal disorder. Due to lack of related endogenous enzymes capable of digesting these two oligosaccharides, more oligosaccharides will be fermented in the hindgut which then cause flatulence and diarrhea [87,88]. Trypsin inhibitor (TI) in soybean meal can bind the trypsin and chymotrypsin secreted by the pancreas and then impair their bioactivity [70]. In raw soybeans, there are two major trypsin inhibitors, including Kunitz trypsin inhibitor (80%) and Bowman–Birk inhibitor (20%) [89]. However, these trypsin inhibitors are heat-labile and normal processing of soybean meal can significantly decrease the activity of these proteins [90]. The majority of commercially prepared heated meals retain as much as 20% of the Bowman–Birk inhibitor of chymotrypsin and trypsin, as well as the Kunitz inhibitor of trypsin [91]. However, the level of TI is not always consistent due to different soybean sources and heating processing [32]. Lectins are anti-nutritional factors because of their ability to bind glycoprotein receptors on the epithelial cells, and the binding can negatively influence the absorption of nutrients in the gut then further impair growth performance [70]. Because lectins are also heat-labile proteins, commercial heating process is considered an effective method to remove them [92].

## 4. Different Processed Soy Products

**Figure 2:**
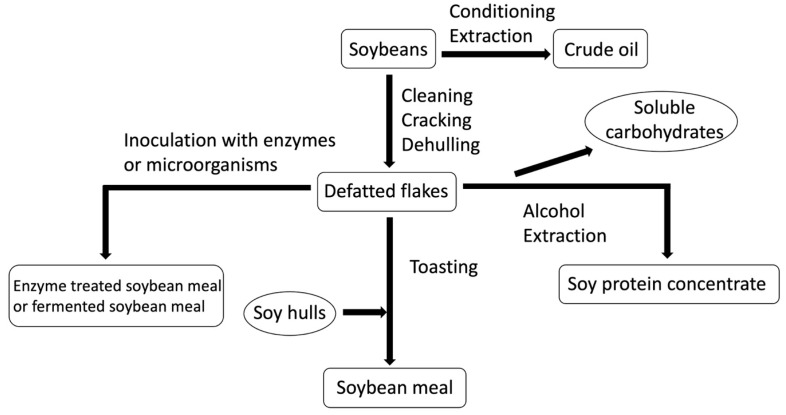
Different technical processing to reduce antinutritional compounds in soybean. The square boxes represent main products during processing and round boxes represent by-products during processing.

Different technical processing has been used to reduce the antinutritional compounds in soybean meal (Figure 2) [32,93,94]. The proximate composition and antinutritional compounds of three commonly processed soy products are listed in Table 1 [29,32,95,96,97,98]. Protein content in all processed soy products were improved and these processes could efficiently remove oligosaccharides in SBM or soy flakes. In addition, SPC and ESBM contain very low allergenic proteins. However, microorganism fermentation shows variance in allergenic proteins including glycinin and β-conglycinin.

### 4.1. Soy Protein Concentrate

Soy protein concentrate is typically prepared by removing soluble carbohydrates from defatted soy flakes [99]. Defatted soy flakes are the remaining parts after oil extraction from soybean; however, in order to avoid a browning reaction, the flakes do not enter the common solemnizer/toaster [32]. Soybean meal, made from defatted soy flakes, is the most extensively known and most widely recognized product. The flakes undergo steam toasting, serving two key purposes: first, to eliminate any residual solvent, and second, to deactivate thermally sensitive antinutritional compounds. Soy protein concentrate preparation commonly involves insolubilization of the protein to remove soluble carbohydrates [100]. There are three common methods to manufacture SPC: aqueous alcohol wash, acid wash, and hot water leaching [99].

The aqueous alcohol process was reported in 1962 and was employed for commercial SPC [101]. Aqueous alcohol (50 to 70%) is used to extract soluble sugars and a small amount of soluble proteins. As a result of the denaturation caused by aqueous alcohol, a significant portion of the proteins lose their solubility and remain with the insoluble polysaccharides. Compared to aqueous alcohol wash, acid wash uses hydrogen chloride to denature protein and extract soluble carbohydrates, which ends up with high protein and less carbohydrates [100]. Hot water leaching is used for SPC production because moist heat is more effective for protein denaturation than dry heat. Elevated-temperature water extraction effectively separates low molecular weight components, such as soluble sugars, from the insoluble proteins [102].

### 4.2. Enzyme-Treated SBM

Enzyme treatment can decrease the allergenicity of glycinin and β-conglycinin in the SBM as well as remove oligosaccharides, such as sucrose, stachyose, and raffinose [103]. In addition, enzyme treatment could improve the content of crude protein and keep more proportions of small peptides, which might be helpful to improve the growth performance of animals [104]. Proteolytic hydrolysis has been shown to eliminate the allergenicity of purified soy proteins [105]. For glycinin, it could be hydrolyzed by pepsin and chymotrypsin, and the fractions less than 20 kDa in hydrolysates are not immunoreactive [106]. For β-conglycinin, protease Proleather GF-F was shown to hydrolyze α and α′ subunits of β-conglycinin, thus reducing the allergenicity [107]. In addition, Alcalase has been shown to effectively reduce the allergenic proteins in SBM by hydrolyzing α′, α and β-subunits of β-conglycinin, and acidic and basic subunits of glycinin [108]. Soy protein hydrolysates containing fragments smaller than 28 kDa have been demonstrated to be non-allergenic, and immune reactivity was observed to have a positive correlation with the stability of glycinin and β-conglycinin [105,109].

Oligosaccharides in SBM, including stachyose and raffinose, cannot be digested in monogastric animals and can accumulate in the large intestine which causes flatus by anaerobic microorganisms [110]. Alpha-galactosidase enzymes are widely found in microorganisms, plants, and animals; it can hydrolyze simple α-D-galactosides and more complex oligosaccharides and polysaccharides [111,112]. Enzymatic hydrolysis of raffinose oligosaccharides in soybean flour led to a significant reduction in stachyose and raffinose levels, with a decrease of 72.3% and 89.2%, respectively, after 6 h of incubation at 40 °C [113]. For commercial products, multiple enzyme combinations are used to reduce the antinutritional compounds in SBM [103].

### 4.3. Fermented SBM

**Figure 3:**
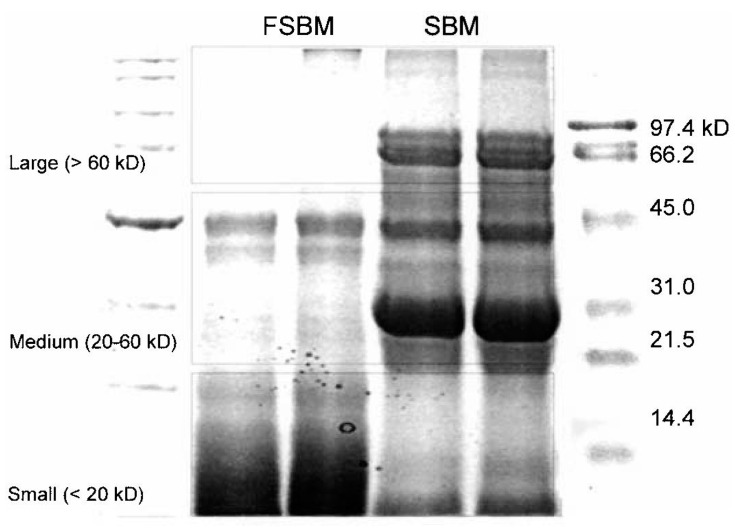
Distribution of peptides in soybean meal and fermented soybean meal (Aspergillus oryzae GB-107). The figure is from Hong et al. [17] and has been used with their permission.

Fermentation is also an effective method to improve the nutritional values for SBM. It not only increases the crude protein content, the proportion of small peptides, free amino acids content, but also decreases degraded anti-nutritional factors, like oligosaccharides, trypsin inhibitors, glycinin and β-conglycinin [18]. Microbial fermentation of SBM is accomplished by utilizing either a fungal or bacterial strain. For fungi-based fermentation, the species of Aspergillus genus are used to ferment SBM [114,115,116]. Fungi-based fermentation reduces TI [114], phytate [117], stachyose and raffinose [97], and significantly increases small-sized peptides (Figure 3) [17]. In addition, it improves approximately 10% of the crude protein content compared to SBM, and most of the essential amino acids are also improved [17,118]. For bacteria-based fermentation, Bacillus spp. and Lactobacillus spp. are used to ferment SBM [119,120]. Much like fungi-based fermentation, it reduces TI, stachyose and raffinose [121], and improves free amino acids and crude protein content [122].

There are some differences between fungi and bacteria fermentation. The soluble protein, in vitro digestibility, antioxidant activity, and the proportion of small-sized peptides in bacteria-based fermentation is higher than fungi-based fermentation [17,122]. This could be attributed to the slower growth of fungi, which leads to a lower population of viable microorganisms [18]. In addition, the parameters of fermentation, such as temperature, time, oxygen level, pH, and inoculum size, play a crucial role in the success of SBM fermentation.

Even though these technical processes improved nutritional values and removed antinutritional compounds in SMB, it also reduces the total amount of bioactive compounds (Table 2) [12,23,29,123,124]. Especially concerning SPC, researchers indicated that aqueous ethanol extraction removes over 95% of total isoflavones [125]. In addition, because of the limited absorption of glucoside isoflavones in SBM, fermentation with β-glycosidases could be an effective method to significantly improve the bioavailability of isoflavones in end soy products [126].

### 4.4. Application of Processed Soy Products

**Figure 4:**
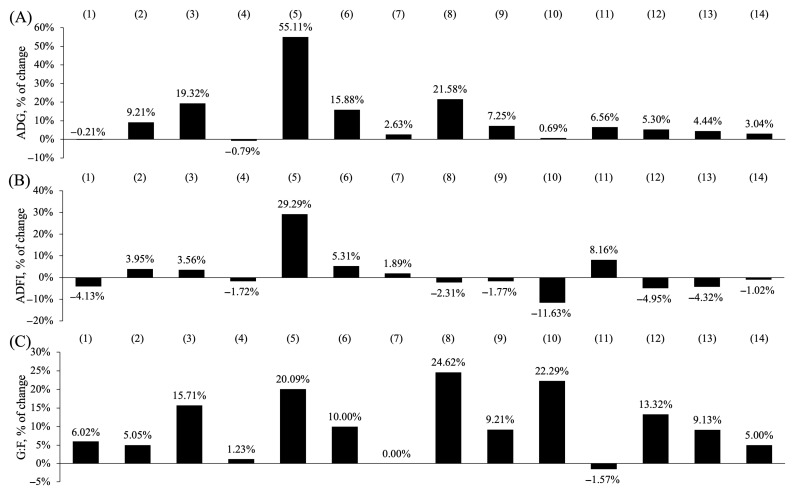
Effects of processed soy products replacing conventional soybean meal on growth performance of young animals. (**A**) represents the change of average daily gain (ADG); (**B**) represents the change of average daily feed intake (ADFI); (**C**) represents the change of gain to feed ratio (G:F). The selected studies (2000 to 2023) were (1) soy protein concentrate (SPC) replacing 100% soybean meal (SBM) in nursery diets [129], (2) SPC replacing 100% SBM in nursery diets [130], (3) SPC replacing 100% SBM in nursery diets [131], (4) SPC replacing 100% SBM in nursery diets [132], (5) enzyme-treated SBM (ESBM) replacing 97% SBM in nursery diets [104], (6) ESBM replacing 50% SBM in phase 1 of nursery diets [98], (7) ESBM replacing 33% SBM in phase 1 of nursery diets [133], (8) ESBM replacing 100% full fat SBM in nursery diets [134], (9) fermented SBM (FSBM) replacing 50% SBM in nursery diets [135], (10) FSBM replacing 28% SBM in phase 1 of nursery diets [31], (11) FSBM replacing 25% SBM in nursery diets [127], (12) FSBM replacing 39% SBM in nursery diets [116], (13) FSBM replacing 45% SBM in nursery diets [128], and (14) FSBM replacing 50% in phase 1 of nursery diets [98].

Due to the high nutrients and low antinutritional compounds in SPC, ESBM, and FSBM, processed soy products have been used to replace conventional SBM with benefits of avoiding deleterious effects of antinutritional compounds in SBM. Studies have been conducted to investigate the effects of the effects of replacing SBM with processed soy products on growth performance of young animals (Figure 4) [31,98,104,116,127,128,129,130,131,132,133,134,135]. The growth performance of young animals was improved as conventional SBM was replaced by SPC [129,130], which could be a result from the reduced allergenic protein content [26]. In addition, trypsin inhibitor is another key antinutritional compound causing reduced growth performance. The use of low-trypsin inhibitor soybean meal improved the growth performance of young animals in relation to increased digestibility of amino acids [136,137]. Results from Zhang et al. also support that SPC had higher standardized ileal digestibility (SID) of amino acids than that of SBM [131]. However, high inclusion of SPC in the nursery diet may negatively affect the feed intake due to palatability issues, thus impairing growth performance [129].

The effects of ESBM replacing conventional SBM on growth performance of young animals were not consistent [98,104,133,138]. Some researchers indicated that the improved growth performance was attributed to the increased nutrient digestibility and some low antinutritional compounds in ESBM [98,104]. Also, due to the higher amount of small-sized peptides in ESBM than SBM, young animals had a considerable capacity to digest it in the small intestine [139]. However, other researchers who observed opposite results indicated that high inclusion of ESBM in the diets could increase water holding capacity and restrict the feed intake of animals [133,140]. The ADFI showed a negative correlation with water holding capacity since higher water holding capacity of diets would limit the transit rate of digesta [141,142]. Due to advantages in ESBM including low antinutritional compounds and high proportion of small-sized peptides, the nutrient digestibility increased compared to conventional SBM [104,143]. By feeding ESBM and replacing SBM, the oxidative status, immune response, and intestinal barrier integrity were improved, which also could be attributed to the low antinutritional compounds in ESBM [98,133].

The inclusion of FSBM replacing conventional SBM in diets improved growth performance of young animals, which was attributed to low antinutritional compounds and a high proportion of small-sized peptides [31,127,135]. Additionally, the metabolites from microorganisms such as lactic acid remained in fermented products and played roles in growth performance improvement [144]. The nutrient digestibility of FSBM was not consistent in the studies [114,130,145]. This inconsistency might be due to the different strains used for fermentation, fermentation conditions, and the drying process [127]. These results also could be explained by the different fermentation processes of FSBM.

## 5. Functional Peptides in Processed Soy Products

Enzymatic hydrolysis and microorganism fermentation could break down soy proteins and produce small peptides [146,147]. Bioactive peptides are specific protein fragments that have a beneficial influence on bodily functions, potentially contributing to overall health. A multitude of soy-derived peptides exhibiting diverse and advantageous physiological effects have been successfully identified, including hypolipidemic, anti-diabetic, anti-cancer, hypotensive, anti-inflammatory, and antioxidant effects [148].

Consumption of soy has consistently demonstrated antiobesity or anorectic properties, exemplified by the impact of soy protein in reducing body weight in obese mice [149]. It is well established that soy protein consumption reduces serum total cholesterol, low-density lipoprotein cholesterol, triglycerides, as well as hepatic cholesterol, which suggested soy protein could alleviate the metabolic syndrome caused by obesity [150]. Soy protein has been indicated to reduce feed intake and increase metabolic rate [151,152]. Previous study suggested that the peptides (Leu-Pro-Tyr-Pro-Arg, Pro-Gly-Pro) from soybean glycinin showed anorectic activities [153]. In addition, several arginine-concentrated fragments in β-conglycinin have been indicated to bind the intestinal cell component, especially the fragment from 51 to 63 of the β subunit, negatively affecting the feed intake of rats via stimulating cholecystokinin (CCK) [154]. In the pig model, soybean protein hydrolysate, especially β-conglycinin hydrolysate, stimulated CCK secretion and inhibited feed intake through calcium-sensing receptors [155]. On one hand, the functional peptides from soy protein showed beneficial effects on obese animals. On another hand, these peptides resulting in feed intake reduction could potentially impair young animals. The reduced feed intake immediately following weaning is accountable for villous atrophy and diminished growth rate in the pig model, which can severely impair the benefits from hog producers [156].

The antioxidant activity of bioactive peptides can be ascribed to their capacity for scavenging free radicals, preventing lipid peroxidation, and chelating metal ions. The radicals scavenging activity of soy protein including glycinin and β-conglycinin undergoes three-to-five folds enhancement following enzymatic digestion [157]. There were six antioxidative peptides that were isolated from β-conglycinin hydrolysate by using Bacillus spp. fermentation, and potent antioxidative peptides within the F2 fraction of fermented soybean protein meal hydrolysate have been successfully isolated and purified through the utilization of *L. plantarum* Lp6 [158,159]. In addition, immunomodulatory peptides were found in soy protein and could boost immune cell functions, such as lymphocyte proliferation, natural killer cell activity, antibody synthesis, and cytokine regulation [160]. A phagocytosis-stimulating peptide has been extracted from trypsin digests of soybean proteins, and it was determined to originate from the α-subunit of β-conglycinin [161]. Lunasin and other lunasin-like peptides, which were purified from defatted soybean flour, demonstrated the ability to mitigate inflammation in LPS-induced macrophages by inhibiting the NF-κB pathway [162]. In an animal experiment, the inclusion of enzyme-treated soy products in nursery diets have been indicated to improve antioxidative status and enhance the health of animals [98,134]. Animals fed with microorganisms fermented with SBM improved their antioxidative capacity and suppressed intestinal inflammation [163,164].

## 6. Conclusions

This review comprehensively introduced SBM and various processed soy products, highlighting their nutritional and bioactive components, such as isoflavones and soyasaponins, which have antioxidant, anti-inflammatory, and antiviral benefits. However, the presence of antinutritional compounds in SBM poses limitations, particularly for young animals. The paper discusses methods like ethanol/acid wash, enzyme treatment, and fermentation to enhance nutritional value while considering potential risks in reducing bioactive compounds. The complex balance required to enhance the nutritional value and preserve the bioactive compounds with antioxidative properties and reduced allergenic proteins in processed soy products was discussed. It emphasizes the necessity of advanced processing techniques to not only enhance the nutrient content but also preserve important bioactive compounds that promote health by competing against oxidative stress and inflammation. This balance is crucial for maximizing the health benefits of soybean products.

## Figures and Tables

**Table 1:** Proximate nutrition composition and antinutritional compounds in processed soy products.

Item	SBM	SPC ^1^			ESBM ^2^			FSBM ^3^		
		SPC1	SPC2	SPC3	ESBM1	ESBM2	ESBM3	FSBM1	FSBM2	FSBM3
Dry matter, %	90.9	93.0	90.6	-	93.5	91.5	93.4	91.3	91.3	90.8
Crude protein, %	41.2	65.0	64.2	-	65	54.4	53.0	53.7	48.7	46.7
Ether extract, %	1.53	1.0	0.1	-	2.5	1.1	2.2	0.8	1.8	1.2
Ash, %	6.1	6.0	-	-	6.8	-	7.6	-	7.2	6.9
Trypsin inhibitor, TIU/mg	1.0–8.0	2.0	-	-	1.0	2.1	0.8	<1.0	0.7	1.9
Glycinin, mg/g	149	<0.1	-	<0.1	<0.1	5.3	0.3	26	12	20.8
β-Conglycinin, mg/g	104	<0.1	-	0.1	<0.1	<0.1	0.2	74	5.8	31.0
Stachyose, %	4.51	2–3	0.86	-	-	0.71	0.13	ND ^4^	0.04	-
Raffinose, %	0.99	0.2–0.3	0.15	-	-	0.16	0.06	ND	0.01	-
Reference	[32,95,96]	[32]	[95]	[29]	[32]	[97]	[98]	[97]	[98]	[96]

^1^ SPC, soy protein concentrate; ^2^ ESBM, enzyme-treated soybean meal; ^3^ FSBM, fermented soybean meal; and ^4^ ND, not detected.

**Table 2:** Amino acid composition and isoflavones in processed SBM.

Item	SBM	SPC ^1^	ESBM ^2^	FSBM ^3^
Essential amino acids, %				
Arg	3.45	4.75	3.95	3.91
His	1.28	1.70	1.41	1.47
Ile	2.14	2.99	2.48	2.61
Leu	3.62	5.16	4.09	4.52
Lys	2.96	4.09	3.20	3.27
Met	0.66	0.87	0.71	0.82
Phe	2.40	3.38	2.78	2.89
Thr	1.86	2.52	2.13	2.24
Trp	0.66	0.81	0.72	0.72
Val	2.23	3.14	2.57	2.88
Isoflavones, mg/kg	2096	115	1080	1277
Reference	[12,23]	[12,23]	[12,124]	[12,124]

^1^ SPC, soy protein concentrate; ^2^ ESBM, enzyme-treated soybean meal; and ^3^ FSBM, fermented soybean meal.

## Data Availability

The data presented in this study are available upon request from the corresponding author.
